# The C-reactive protein–albumin–lymphocyte (CALLY) index in early hospital presentation and intravenous thrombolysis in acute ischemic stroke: A retrospective study

**DOI:** 10.1097/MD.0000000000051419

**Published:** 2026-10-30

**Authors:** Ayşe Fethiye Basa Kalafat, Muhammed Güner, Elif Gizem Olcay, Feyza Karadeniz, Bilal Yeniyurt, Salih Fettahoğlu, Rabia Birsen Tapkan, Hilmi Kaya, Süreyya Tuba Fettahoğlu, Utku Murat Kalafat

**Affiliations:** a Department of Emergency Medicine, University of Health Sciences, İstanbul Kanuni Sultan Suleyman Training and Research Hospital, İstanbul, Turkey; b Department of Emergency Medicine, University of Health Sciences, Haseki Training and Research Hospital, Istanbul, Turkey.

**Keywords:** acute ischemic stroke, CALLY index, early hospital presentation, systemic inflammation, thrombolytic therapy

## Abstract

The C-reactive protein–albumin–lymphocyte (CALLY) index reflects systemic inflammation and immune and nutritional status. We investigated whether admission CALLY was associated with early hospital presentation or receipt of intravenous thrombolysis (IVT) in adults with acute ischemic stroke. This single-center retrospective study included 197 adults with acute ischemic stroke admitted between September 2018 and September 2024. All 98 patients who received IVT were included; 99 non-thrombolyzed controls were selected by computer-generated random sampling from a pool constructed using group-level frequency matching. CALLY was calculated as albumin (g/L) × lymphocyte count (×10^9^/L)/[C-reactive protein (mg/L) × 10]. Early presentation was defined as onset-to-door time ≤4.5 hours. Multivariable logistic regression adjusted for age, sex, and National Institutes of Health Stroke Scale (NIHSS) score assessed associations of ln(CALLY) with early presentation and receipt of IVT. Patients who received IVT had higher NIHSS scores (median, 7 vs 1; *P* < .001) and lower CALLY values (median, 1.37 vs 2.64; *P* = .050); however, the CALLY difference was not significant after multiplicity correction (*q* = 0.122). CALLY was inversely correlated with NIHSS score (Spearman ρ = −0.29; *P* < .001). After adjustment, ln(CALLY) was not associated with receipt of IVT (adjusted odds ratio = 1.34; 95% confidence interval = 0.98–1.82; *P* = .063). Its borderline association with early presentation (adjusted odds ratio = 1.34; 95% confidence interval = 1.02–1.77; *P* = .036) was not consistent across sensitivity analyses. CALLY did not differ significantly between early and late presenters (*P* = .100). Lower CALLY values among patients receiving IVT were largely explained by greater stroke severity. No consistent independent association was found between CALLY and either outcome. These findings do not support using CALLY to guide thrombolytic decision-making.

## 1. Introduction

Stroke is one of the leading causes of long-term disability and mortality worldwide. In the management of acute ischemic stroke (AIS), intravenous thrombolysis (IVT) remains the cornerstone of treatment in eligible patients.^[1]^ Advances in endovascular thrombectomy and contemporary antithrombotic strategies have improved outcomes in selected patient populations.^[2,3]^ The 2026 American Heart Association (AHA)/American Stroke Association guideline, which replaces the 2018 guideline and its 2019 update, recommends alteplase or tenecteplase for eligible patients and bases IVT decisions on the treatment time window together with clinical eligibility, including whether the neurological deficit is disabling.^[4]^ Despite these developments, clinical outcomes vary substantially among patients.

One major contributor to this heterogeneity is the systemic inflammatory response and immune dysregulation following cerebral ischemia. Ischemic stroke triggers a complex immune cascade characterized by central neuroinflammation accompanied by peripheral immunosuppression. This response plays a critical role in secondary brain injury, influencing stroke severity, treatment response, and recovery.^[5–7]^

Serum C-reactive protein (CRP) levels are elevated in a substantial proportion of patients with AIS and reflect both tissue injury and the systemic inflammatory burden.^[8]^ Elevated CRP levels in the early phase of stroke are associated with increased mortality and poor functional outcomes.^[9]^

Given that systemic inflammation is multifaceted and cannot be adequately captured by a single biomarker, composite indices that integrate inflammatory, immune, and nutritional parameters have garnered increasing attention. The C-reactive protein–albumin–lymphocyte (CALLY) index combines these 3 components to provide a more comprehensive assessment of systemic inflammatory and immune status.^[10,11]^

In previous studies, high CRP levels have been interpreted as reflecting active inflammation, low albumin levels as reflecting malnutrition and catabolic stress, and reduced lymphocyte counts as reflecting impaired immune function. Accordingly, a lower CALLY index has been associated with a poorer systemic health profile and increased inflammatory burden.^[12]^ These interpretations were derived from prior literature; the CALLY index itself is a composite laboratory ratio and does not directly measure these biological processes. In the field of stroke, the CALLY index has been associated with stroke risk in hypertensive populations^[13]^ and outcomes after reperfusion therapy. Lu et al reported an inverse association between the CALLY index and unfavorable functional outcomes in 473 patients with AIS treated with endovascular thrombectomy.^[14]^ Pan et al, in 439 patients with AIS, showed using multivariable logistic regression that higher CALLY values were independently associated with a lower risk of hemorrhagic transformation and a better 3-month functional outcome.^[15]^ More recently, Li et al reported that among 477 patients undergoing mechanical thrombectomy, higher CALLY values were associated with lower risks of hemorrhagic transformation, malignant cerebral edema, unfavorable 90-day functional outcomes, and mortality after multivariable adjustment.^[16]^

However, these studies focused on posttreatment clinical outcomes in selected, mostly thrombectomy-treated populations. Whether the admission CALLY index is related to the timing of prehospital arrival or receipt of IVT in routine emergency department practice has not been investigated. Therefore, this study aimed to evaluate, in an exploratory manner, the association of the admission CALLY index with early hospital presentation (≤4.5 hours) and receipt of IVT (secondary outcome) in patients with AIS, taking stroke severity into account.

## 2. Materials and methods

### 2.1. Study design and ethical approval

This study was conducted in accordance with the principles of the Declaration of Helsinki and approved by the Scientific and Ethical Review Board of Kanuni Sultan Süleyman Training and Research Hospital (Approval No: KAEK/2024.10.220). Because of the retrospective design of the study, the requirement for informed consent was waived by the ethics committee. The study had a single-center, retrospective, observational design and was reported in accordance with the Strengthening the Reporting of Observational Studies in Epidemiology guidelines.

### 2.2. Patient selection and study population

All patients aged 18 years or older who presented to the emergency department of Kanuni Sultan Süleyman Training and Research Hospital between September 30, 2018, and September 30, 2024, and were diagnosed with AIS were screened for eligibility (Fig. 1).

**Figure 1.**
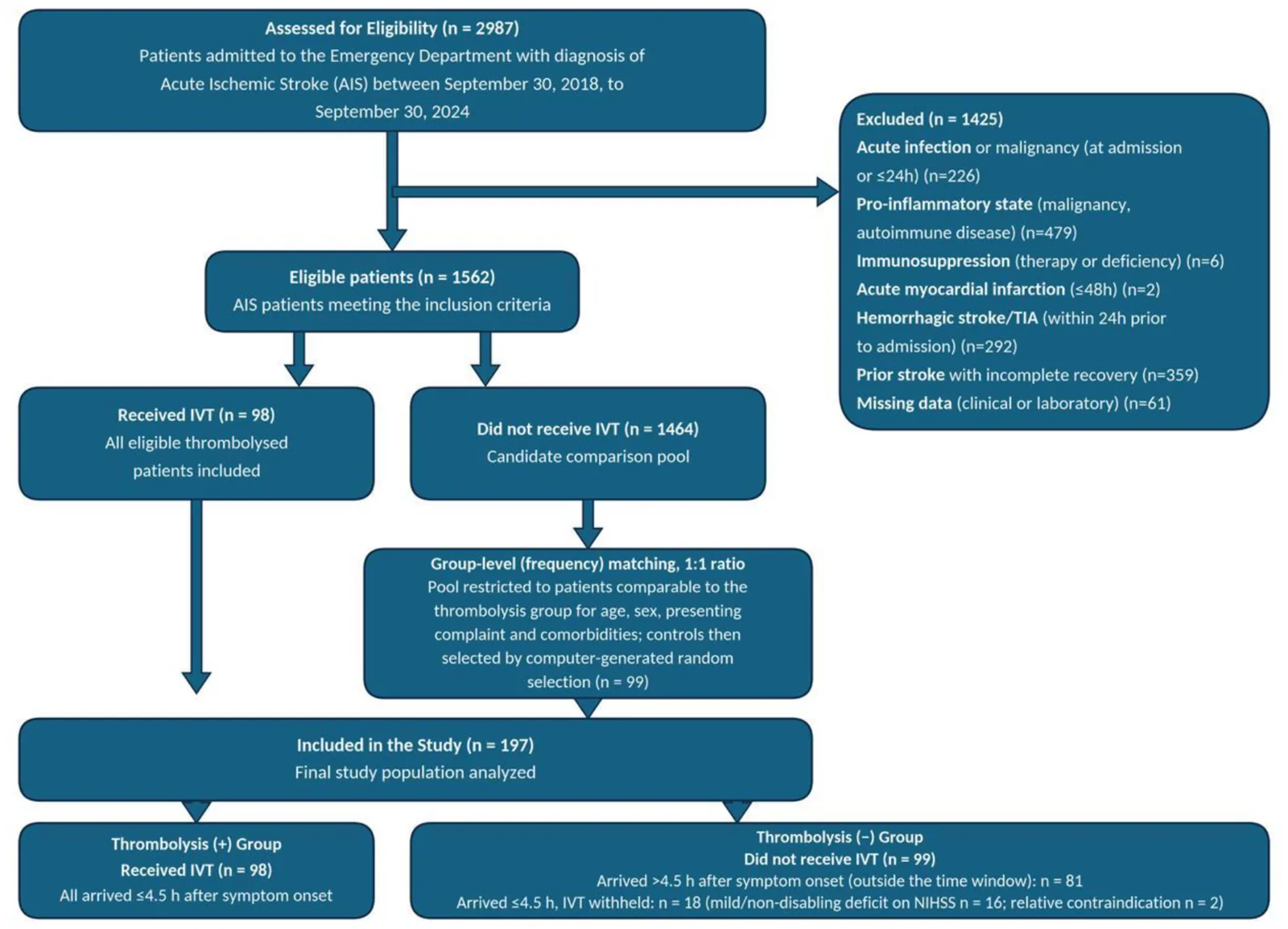
Flowchart of patient selection, group-level (frequency) matching, and study groups. IVT = intravenous thrombolysis, NIHSS = National Institutes of Health Stroke Scale, r-tPA = recombinant tissue plasminogen activator, TIA = transient ischemic attack.

Patients were categorized into 2 groups:

Thrombolytic group: patients who received intravenous recombinant tissue plasminogen activator within 4.5 hours of symptom onset.

Non-thrombolytic group: patients who did not receive thrombolytic therapy. This group comprised patients who arrived more than 4.5 hours after symptom onset and who were therefore outside the IVT time window, and patients who arrived within 4.5 hours but were not treated. Therefore, the groups were defined by receipt of IVT rather than eligibility for IVT.

To limit selection bias, a group-level (frequency) matching strategy with a 1:1 ratio was used rather than individual pair matching. All eligible patients who received IVT during the study period (n = 98) were included. Among the eligible patients who did not receive IVT (n = 1464), the candidate pool was first restricted to patients who were comparable to the thrombolytic group with respect to age, sex, presenting complaint, and comorbidities, and 99 control patients were then selected from this restricted pool by computer-generated random selection. Because controls were not paired with individual thrombolyzed patients, no matched sets were formed, and no caliper was applied; patients who were not randomly selected were not analyzed. The National Institutes of Health Stroke Scale (NIHSS) score and onset-to-door time were deliberately not used as matching variables because they are direct determinants of IVT eligibility (by definition, all thrombolyzed patients arrived within 4.5 hours); matching on them was therefore not feasible and would have precluded the evaluation of early presentation as an outcome. Their influence was addressed by multivariable adjustment and analysis restricted to early presenters.

### 2.3. Clinical assessment and data collection

Detailed medical history was obtained at presentation. Vital signs, capillary blood glucose levels, physical examination findings, and standard 12-lead electrocardiography were recorded in the emergency department as part of routine care. Demographic data, symptom onset time, onset-to-door time, presenting complaints and symptoms (speech disturbance, visual field defect, facial palsy, hemiparesis, hemihypoesthesia, ataxia, and syncope), affected brain regions, and admission NIHSS scores were extracted from the records. For patients who arrived within 4.5 hours but did not receive IVT, the documented reason for nontreatment was also reviewed.

### 2.4. Laboratory parameters and calculation of the CALLY index

Admission laboratory tests included hemoglobin, serum albumin, white blood cell (WBC) count, lymphocyte count, eosinophil count, platelet count, and CRP level. The CALLY index was calculated using the following formula, as applied in recent AIS studies^[14–16]^:


$$\begin{array}{cc} CALLY\;index\\ & =\left[albumin\left(g/L\right)\times lymphocyte\;count\left(\times 10^{9}/L\right)\right]/\left[CRP\left(mg/L\right)\times 10\right]. \end{array}$$

This expression is mathematically equivalent to the original definition of the index, [albumin (g/dL) × lymphocyte count (/μL)]/[CRP (mg/dL) × 10^4^].^[11]^ As a worked example, for a patient with an albumin level of 42.1 g/L, a lymphocyte count of 1.6 × 10^9^/L, and a CRP level of 4.0 mg/L, the CALLY index is (42.1 × 1.6)/(4.0 × 10) = 67.36/40 = 1.68. The same calculation was applied to all the patients.

Thirty-day mortality was also recorded.

### 2.5. Exclusion criteria

Patients were excluded if they had evidence of acute infection or malignancy at admission or within 24 hours, a pro-inflammatory state (malignancy or chronic inflammatory/autoimmune disease), immunosuppression (therapy or deficiency), acute myocardial infarction within 48 hours, hemorrhagic stroke or transient ischemic attack within 24 hours before admission, prior stroke with incomplete neurological recovery, or missing clinical or laboratory data required for the analysis (n = 61). Of the 2987 patients screened, 1425 were excluded, leaving 1562 eligible patients (98 who received IVT and 1464 who did not), from whom the study population of 197 patients was derived as described above (Fig. 1). Because patients with missing data were excluded before group allocation, the analyses were performed on complete cases, and no imputation was undertaken; in the final cohort, only the platelet count of 1 patient was missing.

### 2.6. Thrombolytic treatment protocol

Intravenous thrombolytic therapy was administered according to the AHA guidelines during the study period.^[3]^ Alteplase was administered at a dose of 0.9 mg/kg (maximum 90 mg), with 10% administered as an initial bolus and the remainder infused over 60 minutes.

### 2.7. Outcomes

The primary outcome was early hospital presentation, defined as an onset-to-door time (time from symptom onset to emergency department arrival) of ≤4.5 hours; presentations after 4.5 hours were classified as late. No patient had an onset-to-door time of exactly 4.5 hours. Secondary outcomes included receipt of thrombolytic therapy and 30-day mortality. Because arrival within 4.5 hours is a prerequisite for IVT, the 2 main outcomes overlap substantially; thrombolysis was therefore not treated as an independent determinant of early presentation, and receipt of IVT was additionally analyzed among early presenters only.

### 2.8. Statistical analysis

All statistical analyses were performed using IBM SPSS Statistics version 25.0 (IBM Corp.). The distribution of continuous variables was assessed using the Shapiro–Wilk test, histograms, and skewness. Age, hemoglobin, and platelet count were approximately symmetric and are presented as mean ± standard deviation and compared using the independent samples *t* test. All other continuous variables, including the NIHSS score and the CALLY index, were skewed and are presented as median (interquartile range [IQR]) and compared using the Mann–Whitney *U* test. Categorical variables are presented as n (%) and compared using the Pearson chi-square test or Fisher’s exact test when any expected cell count was <5 (including 30-day mortality). For each comparison, an effect estimate with a 95% confidence interval (CI) was reported: the mean difference, Hodges–Lehmann median difference, or difference in proportions (Newcombe method). To address multiplicity, *P* values within each table were additionally adjusted using the Benjamini–Hochberg false discovery rate procedure (*q* values).

Because controls were selected by group-level frequency matching rather than individual pairing, no matched pairs existed, and conditional (paired) methods were not applicable; therefore, the groups were compared as independent samples, and the matching variables available in the dataset (age and sex) were included as covariates in all multivariable models. Because the CALLY index was highly right-skewed (range = 0.007–169.0), it was natural log-transformed [ln(CALLY)] for the regression analyses. The relationship between the CALLY index and stroke severity was examined using Spearman’s correlation and the Kruskal–Wallis test across NIHSS categories (0–4, 5–15, and >15).

Two multivariable binary logistic regression models were constructed with ln(CALLY) as the main exposure: model 1 with early presentation (≤4.5 hours) as the outcome, and model 2 with receipt of IVT as the outcome. Both models were adjusted for age, sex, and admission NIHSS score, which were selected on clinical grounds. The number of events per variable was at least 24 in both models. Onset-to-door time was not included in model 2 because it perfectly separated the outcomes. In an additional model restricted to early presenters, receipt of IVT was modeled with adjustment for NIHSS because of the small number of non-thrombolyzed patients (n = 18). Sensitivity analyses included expressing CALLY per 1-standard deviation increase in ln(CALLY), modeling CALLY in tertiles, additional adjustment for presenting symptoms (hemiparesis, speech disturbance, and facial palsy), and exclusion of patients with CRP ≥50 mg/L. Results were reported as odds ratios (ORs) with 95% CIs, and discrimination was assessed using the area under the receiver operating characteristic curve (AUC).

No a priori sample size calculation was performed because the sample size was determined by the number of eligible thrombolyzed patients during the study period and an equal number of controls. With 98 and 99 patients per group, the study had 80% power at a two-sided α of 0.05 to detect a standardized between-group difference of 0.40. A two-sided *P* value <.05 was considered statistically significant. The *P* values from the regression and sensitivity analyses were not adjusted for multiplicity; therefore, the regression findings were interpreted as exploratory rather than confirmatory.

## 3. Results

Of the 2987 patients screened, 1425 were excluded, and 1562 were eligible; 197 patients were included in the analysis (Fig. 1). Of these, 42.1% were female (n = 83), and the mean age was 65.6 ± 14.9 years. Ninety-eight patients (49.7%) received intravenous thrombolytic therapy, while 99 (50.3%) did not receive thrombolysis. Of the 99 non-thrombolyzed patients, 81 (81.8%) arrived more than 4.5 hours after symptom onset and were therefore outside the treatment window, and 18 (18.2%) arrived within 4.5 hours. According to the medical records, IVT was not administered to 16 of the 18 patients because their NIHSS-based assessment indicated a mild or non-disabling deficit in the judgment of the treating team and to the remaining 2 patients because of relative contraindications to IVT (median NIHSS of the 18 patients, 1 [IQR 1–3]). Thus, none of the patients in the non-thrombolytic group was considered eligible for IVT by the treatment team.

### 3.1. Comparison of patients with and without thrombolytic therapy

Patients who received thrombolytic therapy presented to the hospital significantly earlier after symptom onset than those who did not receive thrombolysis (median 1.0 hour [IQR 1.0–2.0] vs 24.0 hours [6.0–24.0]; *P* < .001) and had higher NIHSS scores (median 7 [5–10.25] vs 1 [1–3]; Hodges–Lehmann difference 5, 95% CI = 5–6; *P* < .001). Speech disturbances, facial palsy, hemiparesis, and syncope were also more frequent in the thrombolytic group (Table 1). The CALLY index was lower in the thrombolytic group than in the non-thrombolytic group (median 1.37 [IQR 0.69–4.08] vs 2.64 [0.98–5.07]; Hodges–Lehmann difference − 0.52, 95% CI = −1.19 to 0.00; *P* = .050); however, this difference was not significant after correction for multiple comparisons (*q* = 0.122; Table 1, Fig. 2A).

**Table 1:** Comparison of clinical and laboratory characteristics according to receipt of intravenous thrombolysis.

	Thrombolytic therapy		Difference (95% CI)‖	*P* value	*q* value¶
	Did not receive IVT (n = 99)	Received IVT (n = 98)			
Age (yr), M ± SD	64.04 ± 14.77	67.19 ± 15.02	3.15 (−1.03 to 7.34)	.139*	0.198
Female sex, n (%)	39 (39.4)	44 (44.9)	5.5 (−8.2 to 18.9)	.434‡	0.511
NIHSS, Md (IQR)	1 (1–3)	7 (5–10.25)	5 (5 to 6)	**<.001** †	**<0.001**
Hemoglobin level (g/dL), M ± SD	13.18 ± 2.16	13.17 ± 2.01	−0.01 (−0.59 to 0.58)	.985*	0.985
Platelet count (×10^9^/L), M ± SD	240.70 ± 72.38	234.59 ± 70.27	−6.11 (−26.20 to 13.99)	.550*	0.611
Duration of complaint (onset-to-door time, h), Md (IQR)	24.0 (6.0–24.0)	1.0 (1.0–2.0)	−21.5 (−22.0 to −11.0)	**<.001** †	**<0.001**
WBC count (×10^9^/L), Md (IQR)	8.22 (6.14–9.90)	8.40 (7.10–11.07)	0.60 (−0.17 to 1.44)	.126†	0.194
Eosinophil count (×10^9^/L), Md (IQR)	0.17 (0.12–0.28)	0.14 (0.06–0.22)	−0.04 (−0.08 to −0.01)	**.014** †	**0.039**
Lymphocyte count (×10^9^/L), Md (IQR)	2.00 (1.70–2.70)	1.85 (1.38–2.70)	−0.20 (−0.40 to 0.00)	.113†	0.188
Albumin level (g/L), Md (IQR)	39.5 (37.2–42.6)	38.9 (38.0–42.1)	0.0 (−1.1 to 1.2)	.959†	0.985
CRP level (mg/L), Md (IQR)	3.97 (1.40–8.72)	5.32 (2.00–10.00)	0.93 (−0.05 to 2.45)	.061†	0.122
CALLY index, Md (IQR)	2.64 (0.98–5.07)	1.37 (0.69–4.08)	−0.52 (−1.19 to 0.00)	.050†	0.122
Presenting symptoms, n (%)					
Speech disturbance	33 (33.3)	88 (89.8)	56.5 (44.1 to 66.1)	**<.001** ‡	**<0.001**
Facial palsy	17 (17.2)	57 (58.2)	41.0 (27.9 to 52.1)	**<.001** ‡	**<0.001**
Hemiparesis	31 (31.3)	82 (83.7)	52.4 (39.4 to 62.6)	**<.001** ‡	**<0.001**
Hemihypoesthesia	26 (26.3)	38 (38.8)	12.5 (−0.6 to 25.0)	.061‡	0.122
Visual field defect	5 (5.1)	12 (12.2)	7.2 (−0.9 to 15.6)	.072‡	0.131
Ataxia	11 (11.1)	6 (6.1)	−5.0 (−13.4 to 3.2)	.212‡	0.283
Syncope	2 (2.0)	12 (12.2)	10.2 (3.0 to 18.3)	**.005** ‡	**0.017**
30-day mortality, n/N (%)	3/99 (3.0)	0/98 (0.0)	−3.0 (−8.5 to 1.2)	.246§	0.308

CALLY index = [albumin (g/L) × lymphocyte count (×10^9^/L)]/[CRP (mg/L) × 10]. Range (min–max): thrombolysis (−): 0.007 to 169.0; thrombolysis (+): 0.021 to 35.9. The platelet count was missing for 1 patient in the thrombolysis (−) group. Bold *P* and *q* values indicate *P* < .05 and *q* < 0.05, respectively.

Reference range: WBC = 3.8 to 10 × 10^9^/L; platelets = 100 to 400 × 10^9^/L; eosinophil = 0.04 to 0.54 × 10^9^/L; lymphocyte = 0.8 to 4 × 10^9^/L; hemoglobin = 13.0 to 17.5 g/dL; albumin = 39 to 49 g/L; CRP <5 mg/L.

CALLY = C-reactive protein–albumin–lymphocyte index, CI = confidence interval, CRP = C-reactive protein, IQR = interquartile range (25th–75th percentile), IVT = intravenous thrombolysis, M = mean, max = maximum, Md = median, min = minimum, NIHSS = National Institutes of Health Stroke Scale, SD = standard deviation, WBC = white blood cell count.

* Independent samples *t* test.

† Mann–Whitney *U* test.

‡ Pearson chi-square test.

§ Fisher’s exact test (expected cell count <5).

‖ Difference = thrombolysis (+) minus thrombolysis (−): mean difference with 95% CI for normally approximated variables, Hodges–Lehmann median difference with distribution-free 95% CI for skewed variables, and difference in proportions (percentage points) with Newcombe 95% CI for categorical variables.

¶ Benjamini–Hochberg false discovery rate–adjusted *P* value across all comparisons in the table.

**Figure 2.**
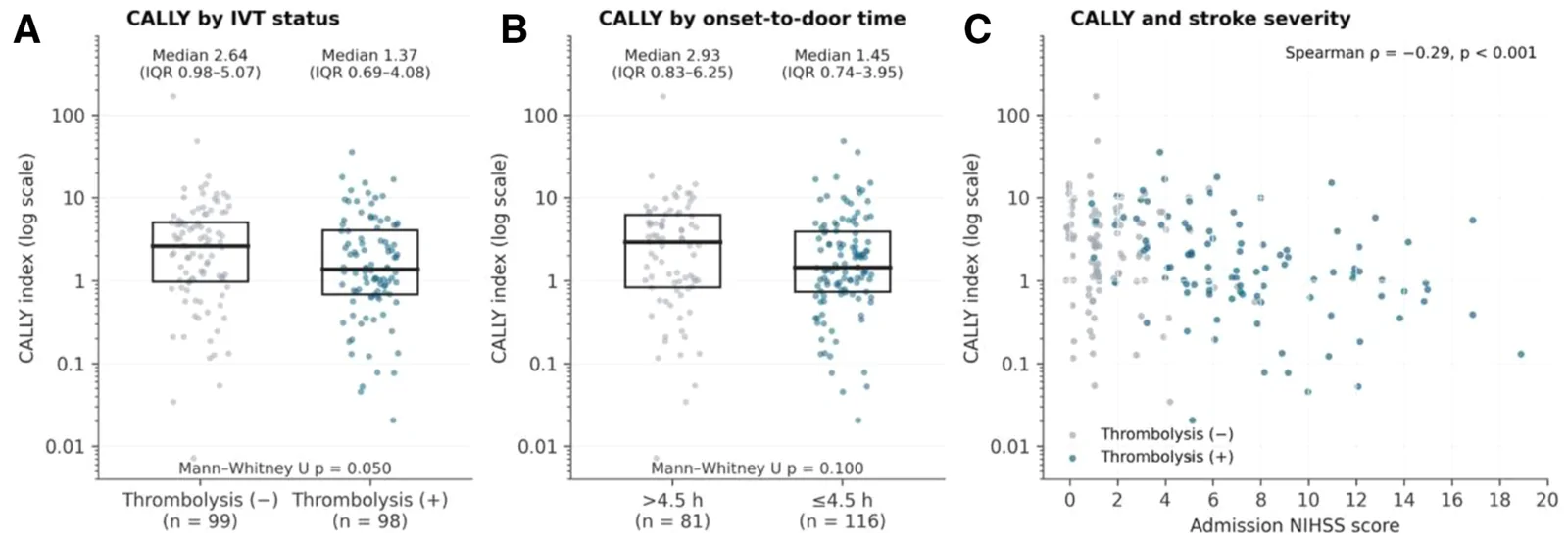
Distribution of the CALLY index (log scale) according to receipt of intravenous thrombolysis (A) and onset-to-door time (B), and its relationship with the admission NIHSS score (C). Boxes show the median and interquartile range; each dot represents 1 patient. CALLY = C-reactive protein–albumin–lymphocyte index, IQR = interquartile range, IVT = intravenous thrombolysis, NIHSS = National Institutes of Health Stroke Scale.

There were no statistically significant between-group differences in age (mean difference = 3.15 years; 95% CI = −1.03 to 7.34; *P* = .139) or sex (female, 44.9% vs 39.4%; *P* = .434), which were the matching variables available in the dataset. The standardized mean differences were 0.21 for age and 0.11 for sex. Although the between-group difference in age was not statistically significant, the SMD of 0.21 suggested some residual imbalance. Age was included as a covariate in all multivariable models. Similarly, hemoglobin levels and WBC, lymphocyte, and platelet counts did not differ significantly between patients who received thrombolytic therapy and those who did not (all *P* > .05).

Eosinophil counts were significantly lower in patients who received thrombolytic therapy (median 0.14 vs 0.17 × 10^9^/L; *P* = .014; *q* = 0.039). Serum albumin levels were comparable between the 2 groups (*P* = .959). CRP levels were numerically higher in patients who received thrombolytic therapy, but this difference was not statistically significant (median 5.32 vs 3.97 mg/L; *P* = .061; Table 1).

Thirty-day mortality occurred in 3 of the 99 patients (3.0%) in the non-thrombolytic group and in none of the 98 patients (0%) in the thrombolytic group; the difference was not statistically significant (difference = −3.0 percentage points, 95% CI = −8.5 to 1.2; Fisher’s exact test *P* = .246). Given the small number of events, this comparison was descriptive.

### 3.2. Comparison of patients presenting within 4.5 hours (≤4.5 hours) of symptom onset and those presenting later (>4.5 hours)

Patients who presented within 4.5 hours of symptom onset (n = 116) were significantly older than those who presented later (n = 81; 67.7 ± 15.0 vs 62.6 ± 14.5 years; *P* = .017; *q* = 0.050) and had markedly higher baseline NIHSS scores (median 6 [IQR 3–9] vs 1 [1–2.5]; *P* < .001; Table 2). Of the early presenters, 98 (84.5%) received IVT, whereas no late presenter did; therefore, early presentation and receipt of IVT overlapped substantially.

**Table 2:** Comparison of patients presenting early (≤4.5 hours) and late (>4.5 hours) after symptom onset.

	Onset-to-door time		Difference (95% CI)‖	*P* value	*q* value¶
	>4.5 hours (n = 81)	≤4.5 hours (n = 116)			
Age (yr), M ± SD	62.59 ± 14.48	67.72 ± 14.96	5.12 (0.91 to 9.34)	**.017** *	0.050
Female sex, n (%)	31 (38.3)	52 (44.8)	6.6 (−7.4 to 19.9)	.359‡	0.423
NIHSS, Md (IQR)	1 (1–2.50)	6 (3–9)	5 (4 to 5)	**<.001** †	**<0.001**
Hemoglobin level (g/dL), M ± SD	13.15 ± 2.30	13.19 ± 1.92	0.04 (−0.56 to 0.63)	.897*	0.944
Platelet count (×10^9^/L), M ± SD	241.85 ± 73.82	234.74 ± 69.54	−7.11 (−27.55 to 13.33)	.493*	0.548
Duration of complaint (onset-to-door time, h), Md (IQR)	24.0 (10.0–48.0)	1.0 (1.0–2.0)	−22.0 (−23.0 to −22.0)	**<.001** †	**<0.001**
WBC count (×10^9^/L), Md (IQR)	8.24 (6.22–9.93)	8.29 (7.02–10.22)	0.40 (−0.35 to 1.21)	.294†	0.368
Eosinophil count (×10^9^/L), Md (IQR)	0.17 (0.11–0.29)	0.15 (0.06–0.23)	−0.04 (−0.07 to −0.01)	**.020** †	0.051
Lymphocyte count (×10^9^/L), Md (IQR)	2.00 (1.70–2.65)	1.90 (1.40–2.70)	−0.10 (−0.40 to 0.10)	.201†	0.269
Albumin level (g/L), Md (IQR)	39.3 (36.8–42.8)	39.2 (38.0–42.1)	0.0 (−1.1 to 1.3)	.999†	0.999
CRP level (mg/L), Md (IQR)	3.97 (1.33–9.58)	5.00 (2.00–9.49)	0.68 (−0.34 to 1.96)	.179†	0.260
CALLY index, Md (IQR)	2.93 (0.83–6.25)	1.45 (0.74–3.95)	−0.48 (−1.36 to 0.08)	.100†	0.181
Received IVT, n (%)	0 (0.0)	98 (84.5)	–	–	–
Presenting symptoms, n (%)					
Speech disturbance	22 (27.2)	99 (85.3)	58.2 (45.2 to 68.2)	**<.001** ‡	**<0.001**
Facial palsy	17 (21.0)	57 (49.1)	28.2 (14.7 to 39.8)	**<.001** ‡	**<0.001**
Hemiparesis	24 (29.6)	89 (76.7)	47.1 (33.5 to 58.2)	**<.001** ‡	**<0.001**
Hemihypoesthesia	22 (27.2)	42 (36.2)	9.0 (−4.3 to 21.5)	.182‡	0.260
Visual field defect	4 (4.9)	13 (11.2)	6.3 (−2.1 to 13.9)	.123‡	0.205
Ataxia	11 (13.6)	6 (5.2)	−8.4 (−17.9 to −0.3)	**.039** ‡	0.086
Syncope	0 (0.0)	14 (12.1)	12.1 (5.5 to 19.2)	**.001** ‡	**0.004**
30-day mortality, n/N (%)	3/81 (3.7)	0/116 (0.0)	−3.7 (−10.3 to 0.3)	.068§	0.136

CALLY index = [albumin (g/L) × lymphocyte count (×10^9^/L)]/[CRP (mg/L) × 10]. Range (min–max): >4.5 hours: 0.007 to 169.0; ≤4.5 hours: 0.021 to 48.6. Early presentation was defined as onset-to-door time ≤4.5 hours; no patient had an onset-to-door time of exactly 4.5 hours. Receipt of IVT was not statistically tested because no patient in the >4.5-hour group received IVT. The platelet count was missing for 1 patient; available-case analysis was used for this variable. Bold *P* and *q* values indicate *P* < .05 and *q* < 0.05, respectively.

Reference range: WBC = 3.8 to 10 × 10^9^/L; platelets = 100 to 400 × 10^9^/L; eosinophil = 0.04 to 0.54 × 10^9^/L; lymphocyte = 0.8 to 4 × 10^9^/L; hemoglobin = 13.0 to 17.5 g/dL; albumin = 39 to 49 g/L; CRP <5 mg/L.

CALLY = C-reactive protein–albumin–lymphocyte index, CI = confidence interval, CRP = C-reactive protein, IQR = interquartile range (25th–75th percentile), IVT = intravenous thrombolysis, M = mean, max = maximum, Md = median, min = minimum, NIHSS = National Institutes of Health Stroke Scale, SD = standard deviation, WBC = white blood cell count.

* Independent samples *t* test.

† Mann–Whitney *U* test.

‡ Pearson chi-square test.

§ Fisher’s exact test (expected cell count <5).

‖ Difference = ≤4.5 hours minus >4.5 hours: mean difference with 95% CI for normally approximated variables, Hodges–Lehmann median difference with distribution-free 95% CI for skewed variables, and difference in proportions (percentage points) with Newcombe 95% CI for categorical variables.

¶ Benjamini–Hochberg false discovery rate–adjusted *P* value across all comparisons in the table.

The duration of symptoms was significantly shorter in patients who presented early than in those who presented later (median 1.0 hour vs 24.0 hours; *P* < .001). Eosinophil counts were lower in the early presenting group (*P* = .020), although this difference was not significant after correction for multiple comparisons (q = 0.051).

In contrast, no significant differences were observed between the 2 groups in terms of WBC count, lymphocyte count, hemoglobin level, platelet count, albumin level, or CRP level (all *P* > .05). The CALLY index did not differ significantly between the early and late presenters (median 1.45 [IQR 0.74–3.95] vs 2.93 [0.83–6.25]; Hodges–Lehmann difference = −0.48, 95% CI = −1.36 to 0.08; *P* = .100; Table 2, Fig. 2B).

### 3.3. CALLY index and stroke severity

The CALLY index was inversely correlated with the admission NIHSS score (Spearman ρ = −0.29, *P* < .001; Fig. 2C). This correlation was present in the thrombolytic group (ρ = −0.41, *P* < .001) but not in the non-thrombolytic group (ρ = −0.11, *P* = .276). The median CALLY values decreased across NIHSS categories (0–4: 2.93, n = 113; 5–15: 1.06, n = 81; >15: 0.39, n = 3; Kruskal–Wallis, *P* < .001). Among the components of the index, CRP was positively correlated with NIHSS (ρ = 0.28, *P* < .001), whereas the correlations between albumin (ρ = −0.15, *P* = .035) and lymphocyte count (ρ = −0.14, *P* = .054) and NIHSS were weak. The CALLY index did not correlate with onset-to-door time (ρ = 0.07, *P* = .304).

### 3.4. Multivariable analyses

In the unadjusted logistic regression, ln(CALLY) was not significantly associated with early presentation (OR = 0.89, 95% CI = 0.73–1.09; *P* = .253) or with IVT (OR = 0.85, 95% CI = 0.70–1.03; *P* = .098; Table 3). After adjustment for age, sex, and NIHSS, the direction of the point estimates was reversed: ln(CALLY) was not associated with receipt of IVT (model 2: adjusted OR [aOR] = 1.34, 95% CI = 0.98–1.82; *P* = .063) and showed a borderline positive association with early presentation (model 1: aOR = 1.34, 95% CI = 1.02–1.77; *P* = .036). The NIHSS score was the dominant independent correlate for both early presentation (aOR = 2.01; 95% CI = 1.63–2.46) and IVT (aOR = 2.43, 95% CI = 1.90–3.11; both *P* < .001). Among early presenters, ln(CALLY) was not associated with IVT after adjustment for the NIHSS (aOR = 1.21, 95% CI = 0.72–2.04; *P* = .472).

**Table 3:** Logistic regression analyses of the association of the CALLY index with early hospital presentation and receipt of IVT.

Analysis	Early presentation (≤4.5 hours)		Receipt of IVT	
	OR (95% CI)	*P* value	OR (95% CI)	*P* value
Primary models, all patients (n = 197; 116 early presenters; 98 thrombolyzed)				
ln(CALLY), per 1-unit increase – unadjusted	0.89 (0.73–1.09)	.253	0.85 (0.70–1.03)	.098
ln(CALLY), per 1-unit increase – adjusted	1.34 (1.02–1.77)	**.036**	1.34 (0.98–1.82)	.063
NIHSS, per 1-point increase (adjusted model)	2.01 (1.63–2.46)	**<.001**	2.43 (1.90–3.11)	**<.001**
Age, per 1-year increase (adjusted model)	1.03 (1.00–1.06)	.084	1.00 (0.97–1.03)	.964
Female sex (adjusted model)	1.02 (0.46–2.29)	.957	0.74 (0.29–1.89)	.531
Restricted to early presenters (n = 116; 98 thrombolyzed, 18 not thrombolyzed)				
ln(CALLY) – unadjusted	–	–	0.76 (0.52–1.13)	.172
ln(CALLY) – adjusted for NIHSS	–	–	1.21 (0.72–2.04)	.472
Sensitivity analyses (adjusted for age, sex, and NIHSS)				
ln(CALLY), per 1-SD increase	1.54 (1.03–2.32)	**.036**	1.54 (0.98–2.42)	.063
ln(CALLY), additionally adjusted for hemiparesis, speech disturbance, and facial palsy	1.37 (1.02–1.85)	**.035**	1.43 (1.02–2.01)	**.039**
CALLY tertile 1 (≤1.01) vs tertile 3 (≥3.49, reference)	0.43 (0.15–1.25)	.120	0.40 (0.12–1.33)	.134
CALLY tertile 2 (1.02–3.39) vs tertile 3 (reference)	1.60 (0.65–3.98)	.309	0.86 (0.30–2.44)	.775
ln(CALLY), excluding patients with CRP ≥ 50 mg/L (n = 191)	1.23 (0.90–1.69)	.194	1.31 (0.90–1.90)	.157
Discrimination, area under the ROC curve (AUC)				
ln(CALLY) alone	0.569		0.581	
NIHSS alone	0.875		0.930	
NIHSS + ln(CALLY)	0.882		0.937	

Binary logistic regression. Adjusted models include age, sex, and admission NIHSS score. The CALLY index was natural log-transformed because of its right-skewed distribution; 1 SD of ln(CALLY) = 1.48. In the analysis restricted to early presenters, adjustment was limited to NIHSS because only 18 patients did not receive thrombolysis. Onset-to-door time was not entered in the thrombolysis models because it perfectly separates the outcome (all thrombolyzed patients arrived ≤4.5 hours). Bold *P* values indicate *P* < .05. No correction for multiple testing was applied to the regression estimates; accordingly, all regression findings should be interpreted as exploratory rather than confirmatory.

CALLY = C-reactive protein–albumin–lymphocyte index, CI = confidence interval, CRP = C-reactive protein, IVT = intravenous thrombolysis, ln = natural logarithm, NIHSS = National Institutes of Health Stroke Scale, OR = odds ratio, ROC = receiver operating characteristic, SD = standard deviation.

In the sensitivity analyses, the adjusted association with early presentation was no longer significant when CALLY was analyzed in tertiles or after exclusion of patients with CRP ≥50 mg/L, and the adjusted association with IVT reached significance only after additional adjustment for presenting symptoms (aOR = 1.43, 95% CI = 1.02–2.01; *P* = .039; Table 3). Thus, neither association was consistent across model specifications. The CALLY index alone showed poor discrimination for IVT (AUC = 0.581) and early presentation (AUC = 0.569), and adding ln(CALLY) to the NIHSS score changed the AUC for IVT only from 0.930 to 0.937.

## 4. Discussion

In this retrospective single-center study, we evaluated the associations of the CALLY index with early hospital presentation and receipt of IVT in patients with AIS. In the unadjusted analyses, patients who received IVT presented earlier, had higher baseline NIHSS scores, and modestly lower CALLY index values. However, the CALLY difference was of borderline significance, did not remain significant after correction for multiple comparisons, and was not statistically significant after adjustment for stroke severity. No consistent association was found between the CALLY index and either early presentation or receipt of IVT, and the index added little discriminatory information beyond the NIHSS score.

The shorter time from symptom onset to hospital arrival and higher baseline NIHSS scores observed among patients receiving thrombolytic therapy were consistent with those of previous studies.^[17,18]^ Patients with more severe clinical presentations are more likely to perceive their symptoms as serious and seek emergency medical care promptly, which increases their likelihood of being candidates for thrombolytic therapy. In contrast, patients with milder neurological deficits may have underestimated the symptom severity, leading to prehospital delays. Lee et al found that a lower initial NIHSS score was associated with late hospital arrival,^[18]^ and a meta-analysis of 30 observational studies identified symptom recognition and perception of symptoms as severe as factors associated with a lower risk of prehospital delay.^[19]^ Similarly, a tertiary-center study from South India reported that patients with more severe strokes were more likely to arrive early, whereas not perceiving symptoms as serious and transport barriers were associated with delays.^[20]^ Our finding that early presenters had higher NIHSS scores is, therefore, confirmatory rather than novel.

Previous studies have associated lower CALLY index values with worse outcomes after reperfusion therapy.^[14–16,21]^ In our cohort, however, the lower crude CALLY index in thrombolyzed patients appears to have largely reflected confounding by stroke severity; the CALLY index was inversely correlated with the NIHSS score, thrombolyzed patients had substantially higher NIHSS scores, and after adjustment for NIHSS, the association was no longer present, and the point estimate reversed direction. Therefore, the CALLY index should not be interpreted as a determinant of or marker for treatment decisions.

One possible explanation for the inverse relationship between the CALLY index and stroke severity, based on previous literature, is the acute stress response following ischemic stroke, which is characterized by the activation of the sympathetic nervous system and the hypothalamic–pituitary–adrenal axis. Increased catecholamine and cortisol release may reduce peripheral lymphocyte and eosinophil counts and may accompany elevations in inflammatory markers, particularly in patients with more severe stroke.^[22–24]^ However, catecholamine, cortisol, and serial inflammatory markers were not measured in the present study, and lymphocyte and albumin levels did not differ significantly between the treatment groups. Therefore, this interpretation should be regarded as hypothesis-generating rather than as a mechanism demonstrated by our data.

Consistent with this caution, the individual components of the index behaved differently in our data: CRP showed the strongest correlation with NIHSS score, whereas the correlations of albumin and lymphocyte count with NIHSS were weak. Early CRP elevation after stroke may reflect the extent of tissue injury,^[8,9]^ but may also be influenced by factors that were not captured in our dataset. Therefore, it cannot be assumed that the composite index captures inflammation, immune status, and metabolic response independently of stroke severity.

Although patients presenting within 4.5 hours of symptom onset had higher NIHSS scores, which is consistent with the close relationship between early presentation and clinical severity,^[18,25]^ no statistically significant association was demonstrated between the CALLY index and early presentation in the unadjusted comparison (*P* = .100). The borderline positive association observed after adjustment for NIHSS was not robust across sensitivity analyses and, given the number of models examined, may represent a chance finding or residual confounding. It should not be interpreted as evidence that the CALLY index influences or is a marker of prehospital arrival time.

The present study differs from previous CALLY studies of AIS in several respects. Lu et al,^[14]^ Pan et al,^[15]^ Li et al,^[16]^ and Zhu et al^[21]^ evaluated the CALLY index as a prognostic marker for hemorrhagic transformation, cerebral edema, recanalization, functional outcome, and mortality, mostly in patients treated with endovascular thrombectomy, using multivariable models with posttreatment outcomes. In contrast, we examined an emergency department population with a comparison group selected by frequency matching, used the admission CALLY index as the exposure, and focused on hyperacute care processes (arrival timing and receipt of IVT) rather than clinical outcomes. In this setting, the CALLY index provides little information beyond stroke severity.

From a clinical perspective, the current AHA/American Stroke Association guidelines base IVT decisions on the time window and clinical eligibility, including whether the neurological deficit is disabling, and recommend alteplase or tenecteplase for eligible patients.^[4]^ Of the 18 early presenters in our cohort who did not receive IVT, 16 had mild or non-disabling deficits, and 2 had relative contraindications, in keeping with this approach. Laboratory indices such as the CALLY index are not part of this decision-making process, and our data do not support any role for the CALLY index in selecting patients for IVT.

## 5. Limitations

This study had several limitations. First, its retrospective and single-center design may have limited the generalizability of the findings. Second, the comparison group was selected from a restricted pool by frequency matching and random selection rather than consecutively, and patients were not individually matched. Residual imbalances in stroke severity and presenting symptoms remained, and selection bias could not be excluded. Third, several variables relevant to IVT decisions and the CALLY index, including blood pressure, glucose, atrial fibrillation, antithrombotic or anticoagulant therapy, pre-stroke functional status (modified Rankin Scale), stroke subtype, imaging findings (e.g., ASPECTS and large-vessel occlusion), and formal contraindications to IVT were not available in the study dataset in a complete and standardized form and could not be included in the models; therefore, residual confounding was possible. Fourth, the reasons for nontreatment among early presenters were retrospectively obtained from medical records. Fifth, no a priori sample size calculation was performed, the number of deaths was very small, and mortality comparison was underpowered. Sixth, laboratory parameters were assessed only at the time of admission, and temporal changes in biomarkers were not evaluated. Finally, the *P* values from the regression and sensitivity analyses were not adjusted for multiplicity, and some estimates differed across model specifications; therefore, the regression findings should be regarded as exploratory rather than confirmatory. Future multicenter prospective studies with consecutive enrollment and comprehensive adjustment are warranted to clarify the relationship between the CALLY index, stroke severity, and hyperacute stroke care.

## 6. Conclusion

In this retrospective single-center study, we evaluated the association between the CALLY index, receipt of IVT, and early hospital presentation in patients with AIS. The lower crude CALLY index observed in patients receiving IVT was of borderline significance, did not persist after correction for multiple comparisons, and was largely explained by the greater stroke severity. No consistent independent association was observed between the CALLY index and either early hospital presentation or receipt of IVT.

Therefore, the CALLY index should not be used as a tool for thrombolytic decision-making. Whether this provides any information beyond stroke severity in the hyperacute phase of AIS should be examined in larger prospective studies.

## Author contributions

**Conceptualization:** Ayşe Fethiye Basa Kalafat, Muhammed Güner, Bilal Yeniyurt, Salih Fettahoğlu, Süreyya Tuba Fettahoğlu, Utku Murat Kalafat.

**Data curation:** Ayşe Fethiye Basa Kalafat, Muhammed Güner, Elif Gizem Olcay, Feyza Karadeniz, Bilal Yeniyurt, Salih Fettahoğlu.

**Formal analysis:** Elif Gizem Olcay, Feyza Karadeniz, Rabia Birsen Tapkan, Hilmi Kaya, Utku Murat Kalafat.

**Investigation:** Ayşe Fethiye Basa Kalafat, Muhammed Güner, Elif Gizem Olcay, Feyza Karadeniz, Bilal Yeniyurt, Salih Fettahoğlu.

**Methodology:** Ayşe Fethiye Basa Kalafat, Rabia Birsen Tapkan, Hilmi Kaya, Utku Murat Kalafat.

**Resources:** Bilal Yeniyurt, Salih Fettahoğlu.

**Software:** Rabia Birsen Tapkan, Hilmi Kaya, Süreyya Tuba Fettahoğlu.

**Supervision:** Utku Murat Kalafat.

**Writing – original draft:** Ayşe Fethiye Basa Kalafat, Muhammed Güner, Bilal Yeniyurt, Rabia Birsen Tapkan, Utku Murat Kalafat.

**Writing – review & editing:** Ayşe Fethiye Basa Kalafat, Muhammed Güner, Elif Gizem Olcay, Feyza Karadeniz, Bilal Yeniyurt, Salih Fettahoğlu, Rabia Birsen Tapkan, Hilmi Kaya, Süreyya Tuba Fettahoğlu, Utku Murat Kalafat.

## References

[1] National Institute of Neurological Disorders and Stroke rt-PA Stroke Study Group. Tissue plasminogen activator for acute ischemic stroke. N Engl J Med. 1995;333:1581–7. 10.1056/NEJM199512143332401 7477192

[2] Goyal M Menon BK van Zwam WH. Endovascular thrombectomy after large-vessel ischaemic stroke: a meta-analysis of individual patient data from five randomised trials. Lancet. 2016;387:1723–31. 10.1016/S0140-6736(16)00163-X 26898852

[3] Powers WJ Rabinstein AA Ackerson T. Guidelines for the early management of patients with acute ischemic stroke: 2019 update to the 2018 guidelines for the early management of acute ischemic stroke: a guideline for healthcare professionals from the American Heart Association/American Stroke Association. Stroke. 2019;50:e344–418. 10.1161/STR.0000000000000211 31662037

[4] Prabhakaran S Gonzalez NR Zachrison KS. 2026 Guideline for the early management of patients with acute ischemic stroke: a guideline from the American Heart Association/American Stroke Association. Stroke. 2026;57:e316–436. 10.1161/STR.0000000000000513 41582814

[5] Iadecola C Buckwalter MS Anrather J. Immune responses to stroke: mechanisms, modulation, and therapeutic potential. J Clin Invest. 2020;130:2777–88. 10.1172/JCI135530 PMC7260029 32391806

[6] Wang H Zhang S Xie L Zhong Z Yan F. Neuroinflammation and peripheral immunity: focus on ischemic stroke. Int Immunopharmacol. 2023;120:110332. 10.1016/j.intimp.2023.110332 37253316

[7] Chan TYH Ma BY Hung TK Wong JSY Lo BWY. Microglial polarization and therapeutic strategies in post-stroke neuroinflammation. Neurol Ther. 2025;14:2277–93. 10.1007/s40120-025-00825-8 PMC12623516 40971139

[8] Di Napoli M Papa F Bocola V. C-reactive protein in ischemic stroke: an independent prognostic factor. Stroke. 2001;32:917–24. 10.1161/01.str.32.4.917 11283392

[9] den Hertog HM van Rossum JA van der Worp HB.; PAIS investigators. C-reactive protein in the very early phase of acute ischemic stroke: association with poor outcome and death. J Neurol. 2009;256:2003–8. 10.1007/s00415-009-5228-x PMC2780652 19609738

[10] Müller L Hahn F Mähringer-Kunz A. Immunonutritive scoring for patients with hepatocellular carcinoma undergoing transarterial chemoembolization: evaluation of the CALLY index. Cancers (Basel). 2021;13:5018. 10.3390/cancers13195018 PMC8508385 34638502

[11] Iida H Tani M Komeda K. Superiority of CRP-albumin-lymphocyte index (CALLY index) as a non-invasive prognostic biomarker after hepatectomy for hepatocellular carcinoma. HPB (Oxford). 2022;24:101–15. 10.1016/j.hpb.2021.06.414 34244053

[12] Pan Y Wu TT Deng CJ. Association between the C-reactive protein-albumin-lymphocyte (CALLY) index and adverse clinical outcomes in CAD patients after PCI: findings of a real-world study. Rev Cardiovasc Med. 2024;25:111. 10.31083/j.rcm2504111 PMC11264017 39076545

[13] Fan X Qing D Zhao J. The relationship between CALLY index and stroke in hypertensive patients: insights from NHANES. Front Nutr. 2025;12:1592641. 10.3389/fnut.2025.1592641 PMC12146181 40491586

[14] Lu Y Zhu X Xu Y Li Y Dai Q Chang X. Lower CALLY index levels indicate higher poor functional outcome risk in acute ischemic stroke patients treated with endovascular thrombectomy. Front Aging Neurosci. 2025;17:1587861. 10.3389/fnagi.2025.1587861 PMC12061938 40353064

[15] Pan Y Liu Z Tu R. The value of the CRP-albumin-lymphocyte index (CALLY index) as a prognostic biomarker in acute ischemic stroke. Sci Rep. 2025;15:13672. 10.1038/s41598-025-97538-7 PMC12012108 40258922

[16] Li J Qing S Zheng L. Association of the C-reactive protein-albumin-lymphocyte index with clinical outcomes after mechanical thrombectomy for acute ischemic stroke. Front Aging Neurosci. 2026;18:1817455. 10.3389/fnagi.2026.1817455 PMC13161055 42130760

[17] Emberson J Lees KR Lyden P.; Stroke Thrombolysis Trialists’ Collaborative Group. Effect of treatment delay, age, and stroke severity on the effects of intravenous thrombolysis with alteplase for acute ischaemic stroke: a meta-analysis of individual patient data from randomised trials. Lancet. 2014;384:1929–35. 10.1016/S0140-6736(14)60584-5 PMC4441266 25106063

[18] Lee EJ Kim SJ Bae J. Impact of onset-to-door time on outcomes and factors associated with late hospital arrival in patients with acute ischemic stroke. PLoS One. 2021;16:e0247829. 10.1371/journal.pone.0247829 PMC7993794 33765030

[19] Zhao Y Xu Y Fang S. Incidence and associated factors of prehospital care-seeking delay in people with acute ischemic stroke: a systematic review and meta-analysis. Neuroepidemiology. 2025;59:727–42. 10.1159/000542765 39701059

[20] Anees A Panicker P Iype T Sreelekha KR. Assessment of onset-to-door time in acute ischemic stroke and factors associated with delay at a tertiary care center in South India. J Neurosci Rural Pract. 2024;15:86–94. 10.25259/JNRP_325_2023 PMC10927050 38476422

[21] Zhu L Jie S Wu S. Ineffective recanalization and complications in patients with acute ischemic stroke receiving endovascular treatment: predictive value of the C-reactive protein-albumin-lymphocyte (CALLY) index. J Inflamm Res. 2025;18:14649–61. 10.2147/JIR.S535881 PMC12554279 41146948

[22] Kim J Song TJ Park JH. Different prognostic value of white blood cell subtypes in patients with acute cerebral infarction. Atherosclerosis. 2012;222:464–7. 10.1016/j.atherosclerosis.2012.02.042 22460048

[23] Cheng Y Ying A Lin Y. Neutrophil-to-lymphocyte ratio, hyperglycemia, and outcomes in ischemic stroke patients treated with intravenous thrombolysis. Brain Behav. 2020;10:e01741. 10.1002/brb3.1741 PMC7507061 32697441

[24] Cain DW Cidlowski JA. Immune regulation by glucocorticoids. Nat Rev Immunol. 2017;17:233–47. 10.1038/nri.2017.1 PMC9761406 28192415

[25] Chen NC Hsieh MJ Tang SC. Factors associated with use of emergency medical services in patients with acute stroke. Am J Emerg Med. 2013;31:788–91. 10.1016/j.ajem.2013.01.019 23465870

